# Immunotherapy in Elderly Patients—Single-Center Experience

**DOI:** 10.3390/cancers16010145

**Published:** 2023-12-27

**Authors:** Maria João Ramos, Ana Sofia Mendes, Raquel Romão, Joana Febra, António Araújo

**Affiliations:** 1 Medical Oncology Department, Centro Hospitalar Universitário de Santo António, 4099-001 Porto, Portugal; u12649@chporto.min-saude.pt (A.S.M.); antonio.araujo@chporto.min-saude.pt (A.A.); 2 Oncology Research Unit, UMIB—Unit for Multidisciplinary Research in Biomedicine, ICBAS—School of Medicine and Biomedical Sciences, Universidade do Porto, 4050-346 Porto, Portugal

**Keywords:** elderly cancer patients, immune checkpoint inhibitors, geriatric oncology, survival, prognosis

## Abstract

**Simple Summary:**

The rising global aging population poses a notable challenge in cancer management due to increased cancer incidence with age. The use of immune checkpoint inhibitors (ICI) in the treatment of different solid tumors presented as a less toxic alternative compared to chemotherapy treatment for elderly patients. However, the age-related remodeling process of the immune system, termed immunosenescence, is hypothesized to impact the efficacy of ICI in the geriatric oncology population. This study provides additional evidence to support the safety and efficacy of ICI in the treatment of elderly cancer patients by using a retrospective, single-center cohort design to analyze survival outcomes and the response rate of 220 patients. Overall, the study challenges the notion of age as a sole determinant for treatment decisions, highlighting the importance of comprehensive geriatric assessments for personalized cancer care in the elderly and the need for further research in this area.

**Abstract:**

Cancer management faces a substantial challenge posed by the aging demographic. Aging is marked by accumulated DNA damage, and this phenomenon is implicated in the process of tumorigenesis. The concept of immunosenescence, postulated to manifest in elderly individuals, is defined by an age-related decline in T cells and a simultaneous elevation in proinflammatory status, leading to a diminished efficacy in response to immunotherapy. Notably, despite the rising prevalence of cancer in the elderly population, their underrepresentation in clinical trials persists. This underscores the unmet need to evaluate the safety and efficacy of cancer treatment in the elderly. This retrospective, single-center cohort study aimed to assess and evaluate the effectiveness and safety of immunotherapy in patients compared to younger individuals with metastatic solid tumors receiving ICI. A total of 220 patients were included, mostly males, with a median age of 64. The proportion of patients ≥ 65 years old was 56.5%. The use of ICI showed no significant differences concerning overall survival (OS) and progression-free survival (PFS) among age groups across different cancer types (melanoma, non-small-cell lung cancer (NSCLC), renal, and bladder cancer; *p* = 0.388). Concerning the response to treatment in renal cancer patients, a significant difference was observed (*p* = 0.041), suggesting a potential negative impact of age on the treatment response. In patients that presented immune-related adverse events (irAEs), oral corticosteroid therapy was marginally associated (*p* = 0.059) with the elderly population. When evaluating the NSCLC population alone (n = 131, 59.5%), our study revealed a strong association between the development of irAEs, patients’ PFS and OS, and the duration of ICI treatment, but not directly correlated with age. The NSCLC elderly population presented a marginally greater number of irAEs, although without statistical significance (*p* = 0.86). ICI maintained efficacy and safety in elderly patients, challenging the notion that age alone should determine treatment decisions. The findings emphasize the necessity of a comprehensive geriatric assessment rather than relying solely on chronological age for personalized cancer treatment in the elderly population. Further prospective studies are needed to better understand immune responses in older adults and derive predictive biomarkers for cancer treatment.

## 1. Introduction

The World Health Organization (WHO) delineates individuals as elderly if they are 60 years or older in developing countries and 65 years or older in developed countries [1]. The global demographic landscape is on the brink of a significant transformation, with the anticipated tripling of the population aged 80 years or older by 2050, soaring from 143 million in 2019 to a projected 426 million [2]. This surge is predominantly fueled by the dual forces of population aging and growth [3]. The escalating burden of cancer incidence and mortality mirrors the profound impact of aging, population expansion, and shifts in the prevalence and distribution of key cancer risk factors, many of which are intertwined with socioeconomic development [3,4,5].

Aging, characterized as a time-dependent decline in the functionality of living organisms, is intricately linked with cancer progression [4,6,7]. Despite research positing aging as a potential tumor-suppressor mechanism, the abnormal behavior of most senescent cells poses significant threats, potentially culminating in tumor development [6]. Accumulated DNA damage, a pivotal driver of senescence, and the events associated with cellular senescence have been implicated in the process of tumorigenesis. Notably, systematic senescence, where the scale gradually increases and affects the entire system, is a prerequisite for the manifestation of senescent phenotypes and age-related diseases, such as malignant tumors [7,8,9].

Proposed by Walford in 1964, immunosenescence is characterized by diminished adaptive immunity, reduced infection resistance, and an increased autoimmune risk [10]. This natural process of immune system aging results in a decline in immune function, thereby influencing various aspects of immune functional networks and increasing the risk of cancer [11,12,13]. The hallmarks of immunosenescence are age-related declines in coping capacity and concurrent increases in proinflammatory status, a phenomenon termed “inflammaging” by Claudio Franceschi in 2000 [14]. The concept of “inflammaging” signifies a systemic state of chronic low-grade inflammation characterized by heightened blood inflammatory markers, serving as a central pillar of the aging process. Factors such as genetics, exercise, nutrition, prior exposure to microorganisms, gender, and human cytomegalovirus infection exert considerable influence on this inflammatory status [14,15,16,17]. Thymic involution stands out as an important change, affecting both innate and adaptive immune systems, with certain immune cell types exhibiting varying degrees of susceptibility [18,19]. 

The tumor response of innate and adaptive immune systems differs between young and elderly individuals, yet the clinical impact and underlying mechanisms remain largely elusive. For instance, T cells, the primary effectors of acquired immunity, undergo substantial aging-related defects that contribute to immune system damage, increased disease susceptibility, and the occurrence of malignant tumors in the elderly [16,20]. Aging emerges as a paramount risk factor for most cancers, with projections indicating that, by 2050, approximately 6.9 million new cancer cases will be diagnosed in individuals aged 80 years or older worldwide, constituting 21.5% of global cases across all age groups [2].

Despite the significant increase in elderly individuals with cancer, their representation in clinical trials remains inadequate, comprising less than 10% of cancer patients over 75 years old [21,22,23]. 

More recently, the development of immune checkpoint inhibitors (ICI) has revolutionized treatment for a larger group of patients with locally advanced/metastatic disease [24]. Two different ICI have been the most investigated: the PD-1/PD-L1 inhibitors and the CTLA-4 inhibitors. ICI are generally considered as safe agents. Notably, when compared with chemotherapy or other systemic treatments, ICI are associated with a low rate of high-grade adverse events (AEs) and, generally, a more tolerable profile, as detected by quality-of-life assessment [24]. For these reasons, the introduction of ICI in the front-line palliative treatment of diverse tumor types led to reconsidering the treatment paradigm in elderly patients.

Lung cancer is predominantly a disease of the elderly. According to the 2023 annual report of the American Cancer Society, about 80% of newly diagnosed NSCLC patients were 65 years of age or older, reflecting the advanced median age of diagnosis (71 years) [25]. 

Consequently, most of the available evidence about elderly patients concerns NSCLC, and meta-analyses have been conducted to assess the safety and efficacy of ICI in these patients. Overall, in the pretreated setting, comparable efficacy was observed in older and younger adults treated with ICI monotherapy using a cut-off of 65 years. However, in a meta-analysis of 12 randomized clinical trials (RCT), OS benefit with mono-immunotherapy was not observed when considering a cut-off of 75 years [26].

Despite this, studies and clinical trials evaluating the effects of host age on ICI outcomes are limited, and the impact of the elderly microenvironment on immunotherapy responses is often overlooked [27,28,29].

Consequently, a comprehensive understanding of ICI tolerance and effects in the elderly remains elusive [29,30,31]. The age-related remodeling process of the immune system, termed immunosenescence, is hypothesized to impact the efficacy and toxicity of ICI in the geriatric oncology population [31,32,33].

This study aimed to assess the effectiveness and safety of immunotherapy in elderly patients (defined by the WHO definition as ≥65 years old) compared to younger individuals (<65 years old) with solid tumors in a palliative care setting.

## 2. Materials and Methods

### 2.1. Patient Population

This was a retrospective, single-center cohort study of patients aged 18 years or older, with histologically or cytologically confirmed metastatic solid tumors (melanoma, renal cancer, NSCLC, and bladder cancer) who received at least one dose of ICI (pembrolizumab, nivolumab, ipilimumab, avelumab, and atezolizumab) as a single therapy, administered intravenously, at Centro Hospitalar Universitário de Santo António from July 2012 to January 2022. Patients with prior ICI therapy and patients with incomplete data were excluded.

### 2.2. Data Collection

Demographic data, such as patients’ age, Eastern Cooperative Oncology Group (ECOG) performance status, diagnosis date, date of widespread disease confirmation, histology type, number of metastasis locations, metastasis location, prior therapeutic regimens, number of doses of ICI, immune-related adverse events (irAEs) graded according to Common Terminology Classification Adverse Events (CTCAE) version 5.0, tumor response based on Response Evaluation Criteria in Solid Tumors version 1.1, progression date, and death date or last follow-up visit, were all retrieved from the patients’ digital health records.

### 2.3. Statistical Analysis

Descriptive statistics were presented as means and standard deviations for normally distributed variables and medians and quartiles otherwise. For categorical variables, frequencies and percentages were presented. Statistical tests included chi-square tests or Fisher’s exact tests for assessing associations between categorical variables. The use of chi-square or Fisher’s exact tests was based on the Cochran rules. Mann–Whitney tests were used to compare continuous variables after checking normality with the Kolmogorov–Smirnov test and histograms.

The objective response rate (ORR) was defined as the percentage of patients who have a confirmed complete response (CR) or partial response (PR) to the treatment. The overall survival (OS) was defined as the time from the initial administration of ICI therapy until death. The progression-free survival (PFS) was defined as the time from the initial administration of ICI therapy until the documentation of tumor progression or death, whichever occurred first. At the end of the follow-up period, patients who were still alive or had not shown progression of their tumor were considered censored in the analysis.

Survival curves were generated by using the Kaplan–Meier product limit method, and differences in OS and PFS were analyzed by stratifying by age (<65 years old/≥65 years old) using the log-rank and Wilcoxon–Peto tests. Univariate and multivariate Cox regression were used to identify factors with potential prognostic significance.

Data were analyzed with SPSS, version 27.0 (IBM Corp., Armonk, NY, USA, 29.0), and *p* < 0.05 was considered significant [34].

## 3. Results

### 3.1. Patients’ Characteristics

A total of 220 patients were studied, mostly males aged from 32 to 85 years old, with a mean age of 64.5 (SD = 10.0). The proportion of patients ≥ 65 years old was 56.5% (n = 122). Table 1 shows the baseline characteristics for the two groups of age.

Regarding gender distribution, a significant difference was observed between age groups. In the younger age group (<65 years old), 34.0% of the patients were females, whereas in the older age group (≥65 years old), this percentage was 21.3% (*p* = 0.036).

When examining the presence of pleural metastasis, another significant difference emerged. In the younger age group, 5.3% of patients had pleural metastasis, whereas in the older age group, a higher percentage, 17.2%, had pleural metastasis (*p* = 0.008). This finding suggests that the likelihood of developing pleural metastasis may be influenced by age. The occurrence of bone metastasis in these patients was significantly associated with age (*p* = 0.030). In the younger group, 27.7% had bone metastasis, while in the older group, 15.6% had this condition. This trend suggests a potential age-related difference in the prevalence of bone metastasis.

In addition to these significant findings discussed above, some non-significant results contributed to improving the comprehension of the study. Smoking history fell short of statistical significance (*p* = 0.091).

The proportion of patients with a history of smoking in the younger group (77.7%) was higher than that in the older group (67.2%). In patients that presented irAEs, oral corticosteroid therapy was marginally associated *(p* = 0.059) with patients ≥ 65 years of age (34.0%), when compared with patients < 65 years of age (16.7%).

The distribution of patients across ECOG scores (0, 1, and 2) was balanced in both age groups.

Concerning cancer type and histology, the analysis did not identify significant age-related differences in the distribution of cancer types (*p* = 0.388) or histology. Whether patients had melanoma, non-small-cell lung cancer (NSCLC), renal cancer, or bladder cancer did not show substantial variations between the two age groups.

Several other clinical factors, including the number of metastasis locations, the specific sites of metastasis (such as lung, lymph nodes, brain, adrenal gland, hepatic, pleural, bone, peritoneal, skin, pancreatic, and renal), the number of ICI, the specific type of ICI treatment (e.g., ipilimumab, pembrolizumab, nivolumab, atezolizumab, and avelumab), the presence of irAEs, their severity, and treatment (supportive care and/or the use of oral or IV corticosteroids), did not demonstrate statistically significant differences between the two age groups.

### 3.2. Treatment Response and Efficacy

The overall median duration of ICI treatment was 6.0 months (Q1 = 2.0, Q3 = 16.0), overall median follow-up time was 14.5 months (Q1 = 5.0, Q3 = 28.0), and overall median progression-free survival was 6.0 months (Q1 = 2.0, Q3 = 18.0). No differences were found between age groups, with *p* = 0.237, *p* = 0.748, and *p* = 0.450, respectively.

A significant result was found for renal cancer (*p* = 0.041). Of the patients below 65 years old, 6 (26.1%) had no response. Seventeen patients aged 65 years or older (73.9%) had no response. No other significant associations were found in other cancer types. The overall association of age groups with ORR, assessed with the chi-square test, was also not significant (*p* = 0.890). Table 2 presents age comparisons stratified by cancer type.

**Figure 1:**
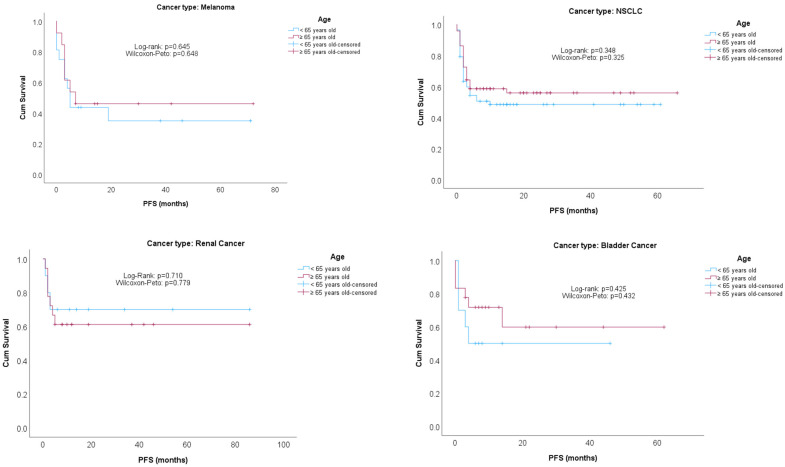
PFS comparisons by age groups, stratified by cancer type.

**Figure 2:**
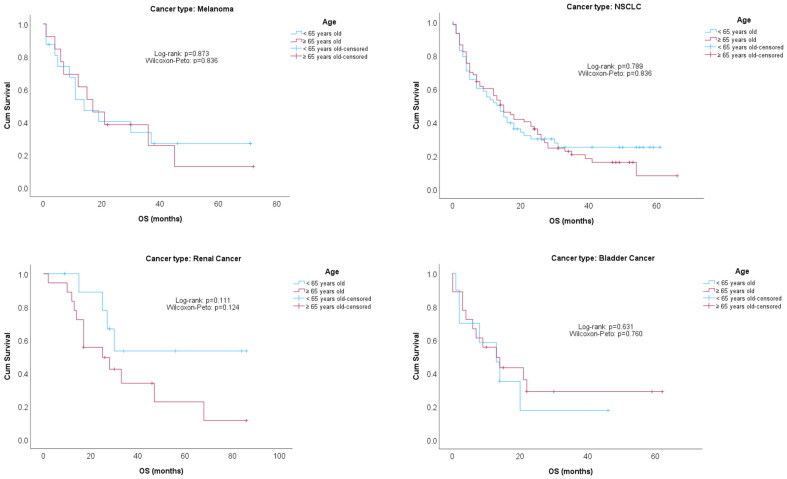
OS comparisons by age groups, stratified by cancer type.

Figure 1 and Figure 2 show Kaplan–Meier survival curves for PFS and OS compared by age groups (<65/≥65), stratified by cancer type. No significant differences were found between age groups, regardless of the cancer type.

Table 3 presents the duration of ICI treatment stratified by cancer type. The duration of ICI treatment was not associated with age groups for each cancer type. The overall age comparison of the duration of ICI treatment, assessed with the Mann–Whitney test, was also not significant (*p* = 0.237).

For our predominantly NSCLC patient population, a separate analysis was conducted. Table 4 and Table 5 showcase the outcomes of multivariate regressions assessing PFS and OS specifically for NSCLC patients. These analyses were adjusted for various factors, including age groups (<65/≥65), ECOG performance status, smoking history, toxicity, treatment duration, histology (non-squamous vs. squamous), and the number of metastasis locations. Concerning PFS (as demonstrated in Table 4), the presence of irAEs emerged as a significant factor affecting PFS (HR = 0.50, 95% CI: 0.28–0.89, *p* = 0.018), and patients experiencing these adverse events seemed to be less likely to progress in their disease.

Regarding OS, Table 5 shows that patients that experienced irAEs were more likely to live longer than those who did not (HR = 0.61 (95% CI: 0.40–0.95, *p* = 0.027)). The duration of ICI treatment (months) was associated with OS (HR = 0.87 (95% CI: 0.84–0.90, *p* < 0.001)), indicating that longer ICI treatment increases the length of survival time.

In contrast, other factors, such as age, gender, ECOG performance status, smoking history, histological subtype, and the number of metastasis locations, did not yield statistically significant associations with PFS or OS, emphasizing toxicity and ICI treatment duration as important determinants of survival in this context.

### 3.3. Immune-Related Adverse Reactions

Overall, the elderly population presented a greater number of irAEs, although without statistical significance (*p* = 0.86). Arthralgia had the highest prevalence in patients aged below 65 years old and pruritus and rash had the highest prevalence among patients aged ≥ 65 years old. Table 6 presents a comparative analysis of toxicity between different age groups.

## 4. Discussion

The rising global aging population poses a notable challenge in cancer management due to increased cancer incidence with age. The introduction of ICI as part of the management of several advanced solid tumors has considerably improved patients’ outcomes. ICI are indeed of paramount importance in the treatment of patients with advanced NSCLC, melanoma, bladder, and renal cancer, as well as other cancer types, and for that reason, efforts are needed to evaluate the impact of aging on the effectiveness of ICI and optimize their use also in the older population [35].

To our knowledge, this study is the first to evaluate the efficacy and safety of ICI in elderly patients beyond NSCLC but also in melanoma, bladder, and renal cancers, and tumors with high prevalence in the older population.

In our study, the use of ICI showed no significant differences concerning OS and PFS among age groups across various cancer types. These findings align with a retrospective study of 410 adult patients with different tumor types (lung, melanoma, and genitourinary), where age did not significantly impact OS or PFS outcomes among those treated with a single-agent ICI. Similarly, irAEs showed no statistically significant disparity between older (≥65 years) and younger patients [31,36]. Notably, a significant difference was observed in the response to treatment among renal cancer patients (*p* = 0.041), suggesting a potential negative impact of age on the treatment response. However, this finding contrasts with existing evidence that indicates no distinct outcomes based on age and could probably be explained by the limited number of patients analyzed [31]. When assessing patients who presented with irAEs, the management of elderly patients showed a marginal association (*p* = 0.059) with oral corticosteroid therapy (34.0%), as opposed to the younger group (16.7%). This could be explained by the fact that elderly individuals often have more comorbidities, necessitating a more cautious approach to toxicity management.

When focusing solely on NSCLC, our study revealed a strong association between the development of irAEs, patients’ PFS and OS, and the duration of ICI treatment, but not directly correlated with age. While several studies have reported the tolerability and efficacy of ICI in NSCLC patients aged ≥ 65, uncertainties persist regarding patients over 75. The scarcity of older patients participating in ICI-related trials, coupled with the increasing prevalence of individuals aged 70 years or older, emphasizes the urgent need for treatment personalization [31]. This calls for a shift toward implementing geriatric assessments rather than solely relying on chronological age for treatment decisions and inclusion criteria in clinical trials.

These observations underscore the need for further investigation to comprehensively understand immune response mechanisms in elderly patients and to identify predictive biomarkers for older adults with cancer [37,38]. Nevertheless, this study has limitations, including its retrospective nature, leading to potential information bias, wide population heterogeneity, and that many patients were primarily treated with anti-PD1 drugs, limiting generalizability to other ICI agents, such as anti-PD-L1 or anti-CTLA-4 agents. Interpretation of the weighted average outcomes should consider these limitations accordingly.

## 5. Conclusions

The transformative impact of immune checkpoint inhibitors on the treatment paradigms for advanced melanoma, NSCLC, bladder, and renal cancer is indisputable.

Age did not affect the efficacy and toxicity of treatment with immunotherapy, suggesting that anticancer immunity remains uncompromised in elderly patients.

The limitations of relying solely on chronological age as a determinant for treatment decisions in the elderly become apparent when considering the heterogeneous nature of this population. Beyond chronological age, our study highlighted the diverse range of molecular and immune alterations present in elderly patients, underscoring the necessity for a more individualized approach to treatment decisions. Recognizing the unmet need to incorporate comprehensive geriatric assessments into daily practice, further prospective studies with a focus on geriatric assessment are warranted to optimize therapeutic strategies in this prevalent and heterogeneous population.

## Figures and Tables

**Table 1:** Patients’ baseline characteristics.

Variable	<65 Years Old	≥65 Years Old	*p*-Value
Gender			
Female	32 (34.0%)	26 (21.3%)	**0.036 (a)**
Male	62 (66.0%)	96 (78.7%)	
Smoking history			
No	21 (22.3%)	40 (32.8%)	0.091 (a)
Yes	73 (77.7%)	82 (67.2%)	
ECOG			
0	33 (35.1%)	32 (26.2%)	0.283 (a)
1	57 (60.6%)	81 (66.4%)	
2	4 (4.3%)	9 (7.4%)	
Cancer type			
Melanoma	16 (17.0%)	13 (10.7%)	0.388 (a)
NSCLC	58 (61.7%)	73 (59.8%)	
Renal Cancer	10 (10.6%)	18 (14.8%)	
Bladder Cancer	10 (10.6%)	18 (14.8%)	
Number of metastasis locations			
1	56 (59.6%)	75 (61.5%)	0.277 (a)
2	26 (27.7%)	39 (32.0%)	
≥3	12 (12.8%)	8 (6.6%)	
Metastasis location			
Lung			
No	50 (53.2%)	71 (58.2%)	0.462 (a)
Yes	44 (46.8%)	51 (41.8%)	
Lymph node			
No	60 (63.8%)	70 (57.4%)	0.337 (a)
Yes	34 (36.2%)	52 (42.6%)	
Brain			
No	85 (90.4%)	113 (92.6%)	0.562 (a)
Yes	9 (9.6%)	9 (7.4%)	
Adrenal gland			
No	86 (91.5%)	115 (94.3%)	0.427 (a)
Yes	8 (8.5%)	7 (5.7%)	
Hepatic			
No	82 (87.2%)	111 (91.0%)	0.376 (a)
Yes	12 (12.8%)	11 (9.0%)	
Pleural			
No	89 (94.7%)	101 (82.8%)	**0.008 (a)**
Yes	5 (5.3%)	21 (17.2%)	
Bone			
No	68 (72.3%)	103 (84.4%)	**0.030 (a)**
Yes	26 (27.7%)	19 (15.6%)	
Peritoneal			
No	93 (98.9%)	119 (97.5%)	0.634 (b)
Yes	1 (1.1%)	3 (2.5%)	
Skin			
No	92 (97.9%)	122 (100.0%)	0.188 (b)
Yes	2 (2.1%)	0 (0.0%)	
Pancreatic			
No	93 (98.9%)	121 (99.2%)	>0.990 (b)
Yes	1 (1.1%)	1 (0.8%)	
Renal			
No	94 (100.0%)	121 (99.2%)	>0.990 (b)
Yes	0 (0.0%)	1 (0.8%)	
Number of ICI lines			
1	51 (54.3%)	56 (45.9%)	0.443 (a)
2	41 (43.6%)	61 (50.0%)	
≥3	2 (2.1%)	5 (4.1%)	
ICI			
Ipilimumab			
No	87 (92.6%)	120 (98.4%)	**0.043 (b)**
Yes	7 (7.4%)	2 (1.6%)	
Pembrolizumab			
No	48 (51.1%)	50 (41.0%)	0.140 (a)
Yes	46 (48.9%)	72 (59.0%)	
Nivolumab			
No	62 (66.0%)	82 (67.2%)	0.846 (a)
Yes	32 (34.0%)	40 (32.8%)	
Atezolizumab			
No	87 (92.6%)	115 (94.3%)	0.613 (a)
Yes	7 (7.4%)	7 (5.7%)	
Avelumab			
No	92 (97.9%)	121 (99.2%)	0.581 (b)
Yes	2 (2.1%)	1 (0.8%)	
Toxicity			
No	52 (55.3%)	72 (59.0%)	0.586 (a)
Yes	42 (44.7%)	50 (41.0%)	
Toxicities (n = 42)			
Toxicity ≥ G3			
No	34 (81.0%)	39 (78.0%)	0.728 (a)
Yes	8 (19.0%)	11 (22.0%)	
Supportive care			
No	9 (21.4%)	15 (30.0%)	0.351 (a)
Yes	33 (78.6%)	35 (70.0%)	
Oral corticosteroid			
No	35 (83.3%)	33 (66.0%)	0.059 (a)
Yes	7 (16.7%)	17 (34.0%)	
IV corticosteroids			
No	37 (88.1%)	42 (84.0%)	0.574 (a)
Yes	5 (11.9%)	8 (16.0%)	

Results are presented as n (%); *p*-values were computed with (a) chi-square tests or (b) Fisher’s exact tests when the Cochran rules were not met.

**Table 2:** Comparison of treatment responses among age groups stratified by cancer type.

Cancer Type	Age Groups	ORR = No Response	ORR = Response	*p*-Value
Melanoma	<65 years old (n = 16)	14 (56.0%)	2 (50.0%)	>0.990 (b)
	≥65 years old (n = 13)	11 (44.0%)	2 (50.0%)	
NSCLC	<65 years old (n = 58)	41 (46.1%)	17 (40.5%)	0.548 (a)
	≥65 years old (n = 73)	48 (53.9%)	25 (59.5%)	
Renal Cancer	<65 years old (n = 10)	6 (26.1%)	4 (80.0%)	**0.041 (b)**
	≥65 years old (n = 18)	17 (73.9%)	1 (20.0%)	
Bladder Cancer	<65 years old (n = 10)	6 (33.3%)	4 (40.0%)	>0.990 (b)
	≥65 years old (n = 18)	12 (66.7%)	6 (60.0%)	

Results are presented as n (%); *p*-values were computed with (a) chi-square tests or (b) Fisher’s exact tests when the Cochran rules were not met.

**Table 3:** Duration of ICI treatment, stratified by cancer type.

Cancer Type	Age Groups	Median (Q_1_–Q_3_)	*p*-Value
Melanoma	<65 years old (n = 16)	7.0 (2.0–22.0)	0.983
	≥65 years old (n = 13)	7.0 (4.0–11.0)	
NSCLC	<65 years old (n = 58)	4.0 (1.0–15.0)	0.277
	≥65 years old (n = 73)	4.0 (2.0–16.0)	
Renal Cancer	<65 years old (n = 10)	12.5 (4.0–28.0)	0.759
	≥65 years old (n = 18)	10.0 (4.0–19.0)	
Bladder Cancer	<65 years old (n = 10)	4.0 (1.0–8.0)	0.286
	≥65 years old (n = 18)	8.5 (3.0–21.0)	

Results are presented as median (Q_1_–Q_3_); *p*-values were computed with Mann–Whitney tests.

**Table 4:** Multivariate Cox regression for PFS in NSCLC.

	B (SE)	HR	95% CI for HR	*p*-Value
Gender (male)	0.16 (0.40)	1.17	0.54–2.56	0.691
Age ≥ 65	−0.41 (0.31)	0.66	0.36–1.22	0.184
ECOG = 0				
ECOG = 1	−0.62 (0.41)	0.54	0.24–1.20	0.132
ECOG = 2	−0.06 (0.67)	0.95	0.25–3.52	0.934
Smoking history (yes)	−0.23 (0.55)	0.79	0.27–2.34	0.675
Toxicity (yes)	−0.70 (0.30)	0.50	0.28–0.89	0.018
Duration of ICI treatment (months)	−0.46 (0.08)	0.63	0.54–.74	<0.001
Histology = Adenocarcinoma	−0.08 (0.39)	0.93	0.43–2.00	0.848
Histology = Other	0.00 (0.72)	1.00	0.24–4.15	0.995
Number of metastasis locations = 1				
Number of metastasis locations = 2	0.10 (0.32)	1.11	0.60–2.06	0.744
Number of metastasis locations ≥ 3	0.24 (0.45)	1.28	0.53–3.07	0.585

**Table 5:** Multivariate Cox regression for OS in NSCLC.

	B (SE)	HR	95% CI for HR	*p*-Value
Gender (male)	−0.27 (0.29)	0.76	0.43–1.35	0.352
Age ≥ 65	0.30 (0.23)	1.35	0.85–2.12	0.201
ECOG = 0				
ECOG = 1	0.36 (0.33)	1.44	0.75–2.74	0.274
ECOG = 2	0.95 (0.55)	2.58	0.87–7.64	0.087
Smoking history (yes)	0.46 (0.40)	1.58	0.72–3.48	0.252
Toxicity (yes)	−0.49 (0.22)	0.61	0.40–0.95	0.027
Duration of ICI treatment (months)	−0.14 (0.02)	0.87	0.84–0.90	<0.001
Histology = Adenocarcinoma	−0.04 (0.27)	0.96	0.56–1.64	0.885
Histology = Other	−0.72 (0.58)	0.49	0.16–1.51	0.212
Number of metastasis locations = 1				
Number of metastasis locations = 2	0.30 (0.24)	1.35	0.85–2.15	0.209
Number of metastasis locations ≥ 3	0.86 (0.36)	2.36	1.17–4.77	0.016

**Table 6:** Immune-related adverse events stratified by age groups.

	<65 Years Old	≥65 Years Old
Hypothyroidism	7 (7.4%)	6 (4.9%)
Pruritus	9 (9.6%)	11 (9.0%)
Rash	6 (6.4%)	11 (9.0%)
Myalgia	7 (7.4%)	4 (3.3%)
Arthralgia	10 (10.6%)	7 (5.7%)
Pneumonitis	3 (3.2%)	4 (3.3%)
Colitis	1 (1.1%)	6 (4.9%)
Bullous pemphigus	0 (0.0%)	1 (0.8%)
Vasculitis	0 (0.0%)	1 (0.8%)
Herpes Zoster	0 (0.0%)	1 (0.8%)
Hepatitis	3 (3.2%)	4 (3.3%)
Nephritis	1 (1.1%)	3 (2.5%)
Hyperthyroidism	4 (4.3%)	0 (0.0%)
Diarrhea	1 (1.1%)	1 (0.8%)
Inflammatory myopathy	1 (1.1%)	0 (0.0%)
Proteinuria	0 (0.0%)	1 (0.8%)
Vitiligo	1 (1.1%)	0 (0.0%)
Cholangitis	1 (1.1%)	1 (0.8%)
Pancytopenia	1 (1.1%)	0 (0.0%)
Fever	1 (1.1%)	2 (1.6%)
Astenia	0 (0.0%)	2 (1.6%)

## Data Availability

The datasets generated and/or analyzed during the current study are not publicly available. However, they are available from the corresponding author upon reasonable request.
